# Supplementary material for: The psychophysics of human three-dimensional active visuospatial problem-solving
Source: Sci Rep. 2023 Nov 15;13:19967. doi: 10.1038/s41598-023-47188-4 (PMC10651907; doi:10.1038/s41598-023-47188-4)
Supplement: Supplementary file 1 — Supplementary Information. [file 41598_2023_47188_MOESM1_ESM.pdf]

The Psychophysics of Human Three-Dimensional Active Visuospatial Problem-Solving – *Supplementary material*

Markus D. Solbach[a,*] **and John K. Tsotsos**[a]

[a]Department of Electrical Engineering and Computer Science, York University, Toronto, ON, Canada M3J 1P3
[*]solbach@yorku.ca

Supplementary Material

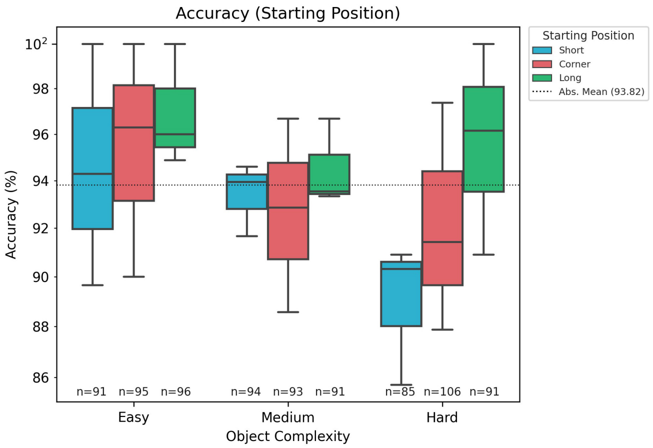

(a)

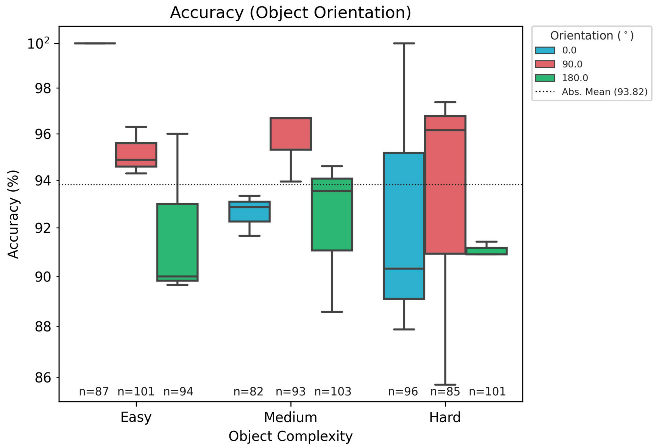

(b)

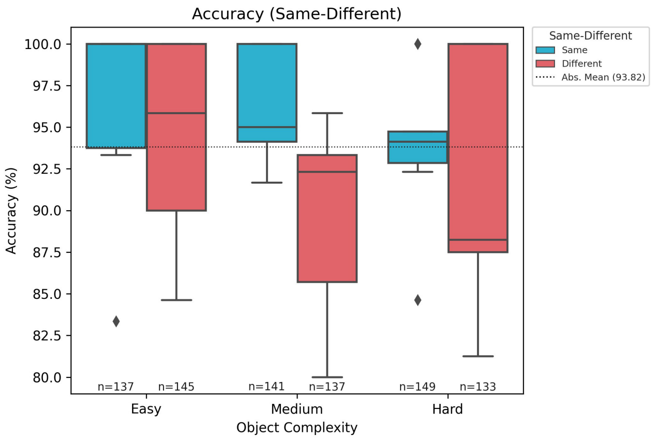

(c)

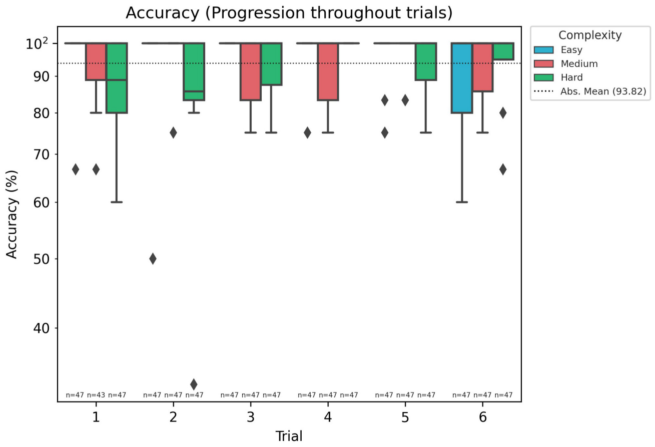

(d)

Figure S1. Plots illustrating the accuracy measured against different experimental variables. **(a)** The effect of the starting position across the different object complexities. The best accuracy for C_e (easy) is from P_c. For C_m the highest accuracy was achieved starting from P_s. Lastly, C_h was most accurately answered starting from P_l, meaning that every complexity class had its unique starting position from which it performed best on average. **(b)** The effect of object orientation. For C_e cases, a clear gradient of accuracy following the increase of orientation is visible. Notably, all trials with C_e with 0° orientation were always answered correctly. This is the only case where this is true. However, for the other complexity levels, a different pattern can be identified; 90° was most accurately identified with 94.82% and 90% for C_m and C_h, respectively. 0° and 180° ranked second and third. Interestingly, the object orientation does not have a significant effect on the accuracy. **(c)** The effect of object sameness. Accuracy is not significantly affected. If the objects are the same; generally, a higher accuracy is seen (94.3%) than for different pairings (91.6%). Lastly, the progression throughout trials **(d)** revealed that no improvement in accuracy appeared – no significant learning effect was observed.

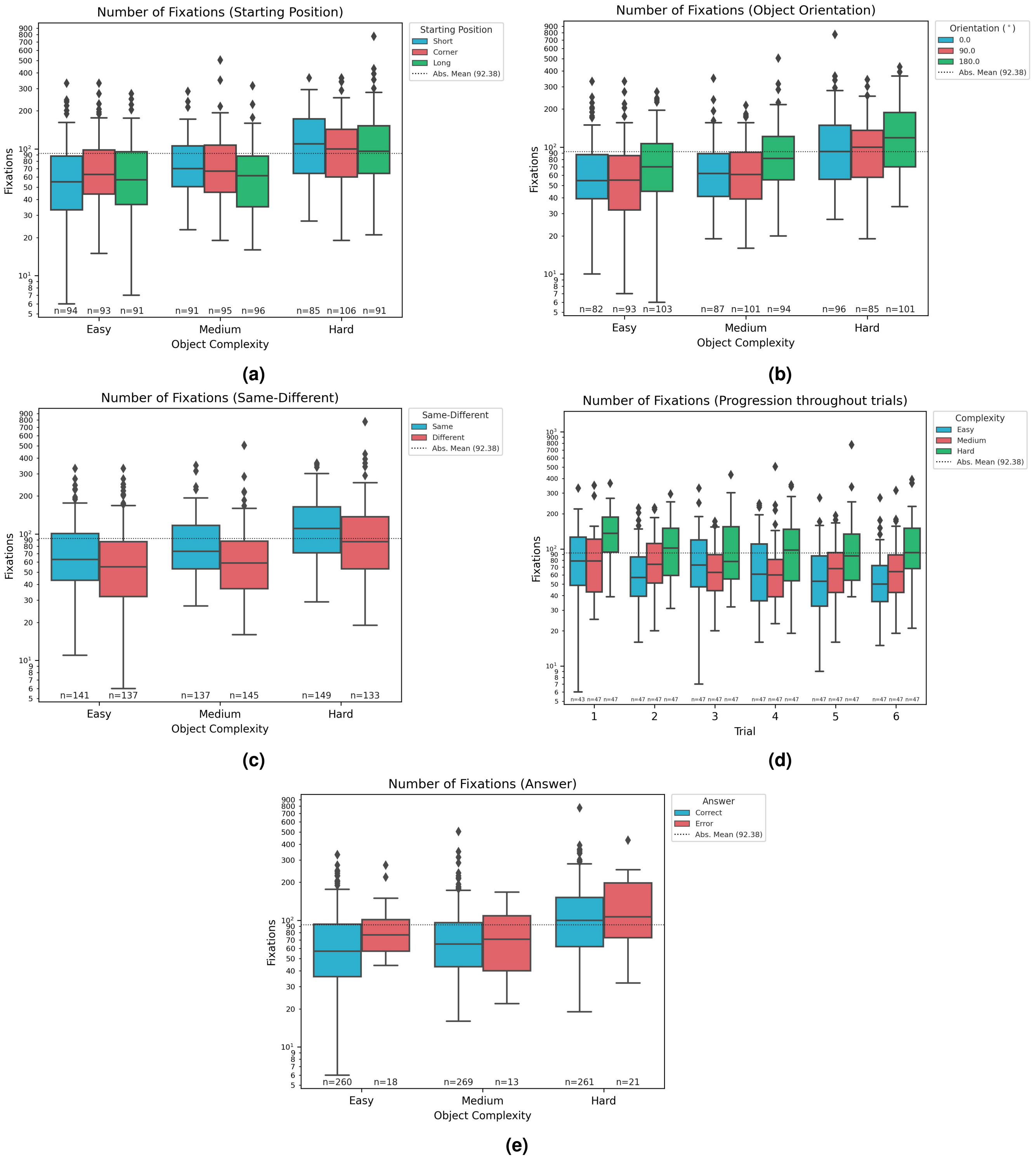

Figure S2. The number of fixations against different experimental variables. **(a)** The effect of the starting position. For C_m and C_h cases, starting from P_s resulted in the most fixations on average, followed by starting from the P_c and P_l. **(b)** The effect of object orientation. Orientations 0° and 90° are similar, varying only a few fixations for the median and upper and lower quartile. In terms of absolute values, a few trials of C_h and orientation of 0° required about 800 fixations. Notably, these trials started from P_l. Larger orientation differences required significantly more fixations regardless of object complexity. **(c)** The evaluation of sameness against the number of fixations. The same pairings always required significantly more fixations than different pairings. The same pairings needed at least 10, in some cases up to 20, fixations on average more. Additionally, error responses required significantly more fixations than correct answers. **(d)** A significant learning effect with respect to the number of fixations is observed. **(e)** The effect of correctness. Error responses result in more fixations than correct answers.

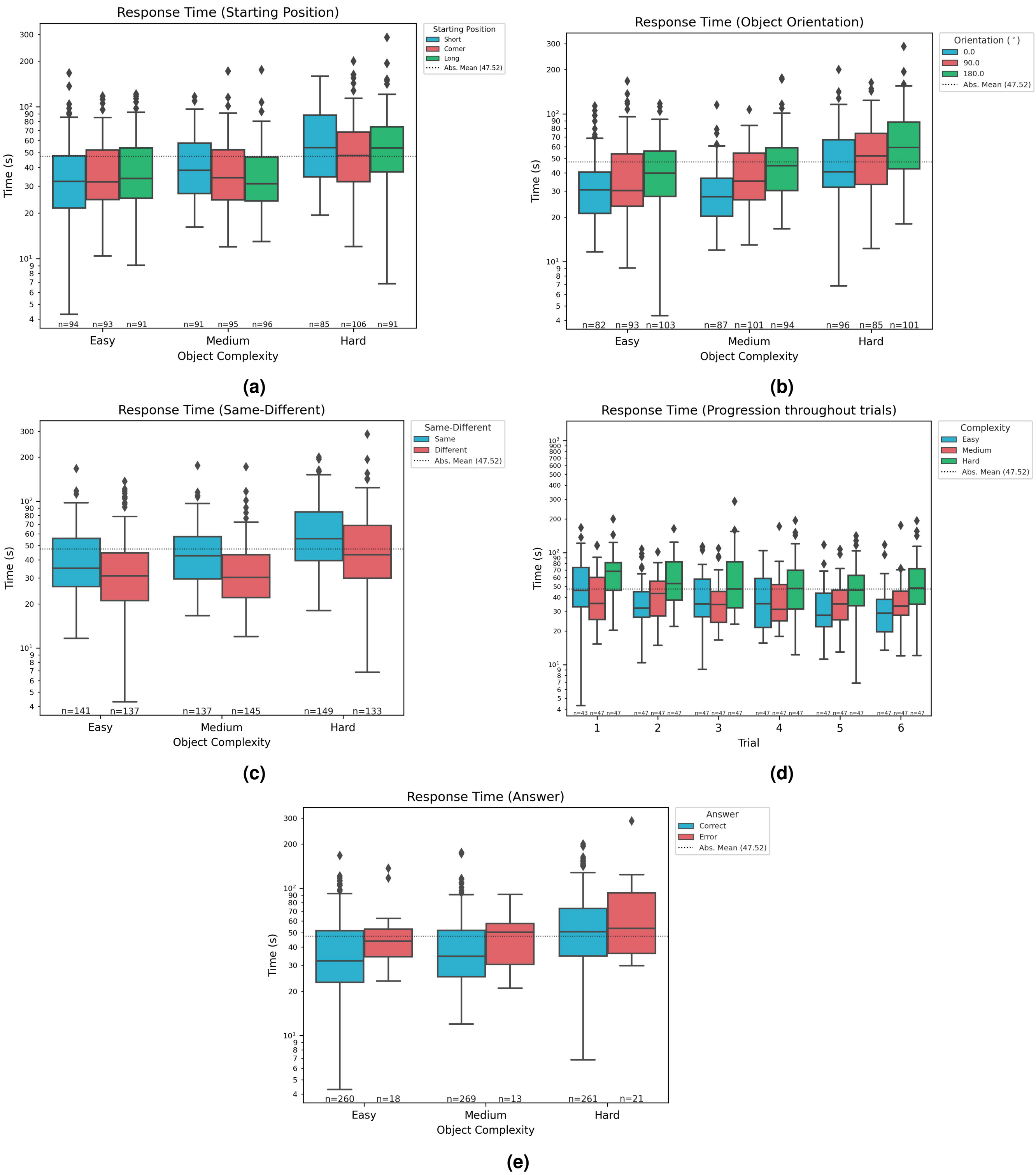

Figure S3. Response Time against different experimental variables. **(a)** Consistent with other measures, the response time is not significantly affected by the starting position. **(b)** The object orientation, however, does affect response time. In general, a smaller orientation difference also means a quicker response time – 0° was answered the quickest, followed by 90°, and 180°. **(c)** The sameness of the stimuli has a distinct effect on the response time – same cases take significantly longer than different ones. **(d)** Subjects seem to develop more efficient strategies with increasing trials completed. Starting at about 47s (Median) at the first trial, the response time drops to about 34s (Median) for trials two to four and drops further to 29s Median at trials five and six. For C_h cases, a drop from the first trial (70s Median) to the second trial (about 50s Median) can be seen. Overall, looking at the impact of progressing trials and their response time, a significant effect is noticed. **(e)** The effect of error/correct answers with respect to the response time: Error answers result in longer response times.

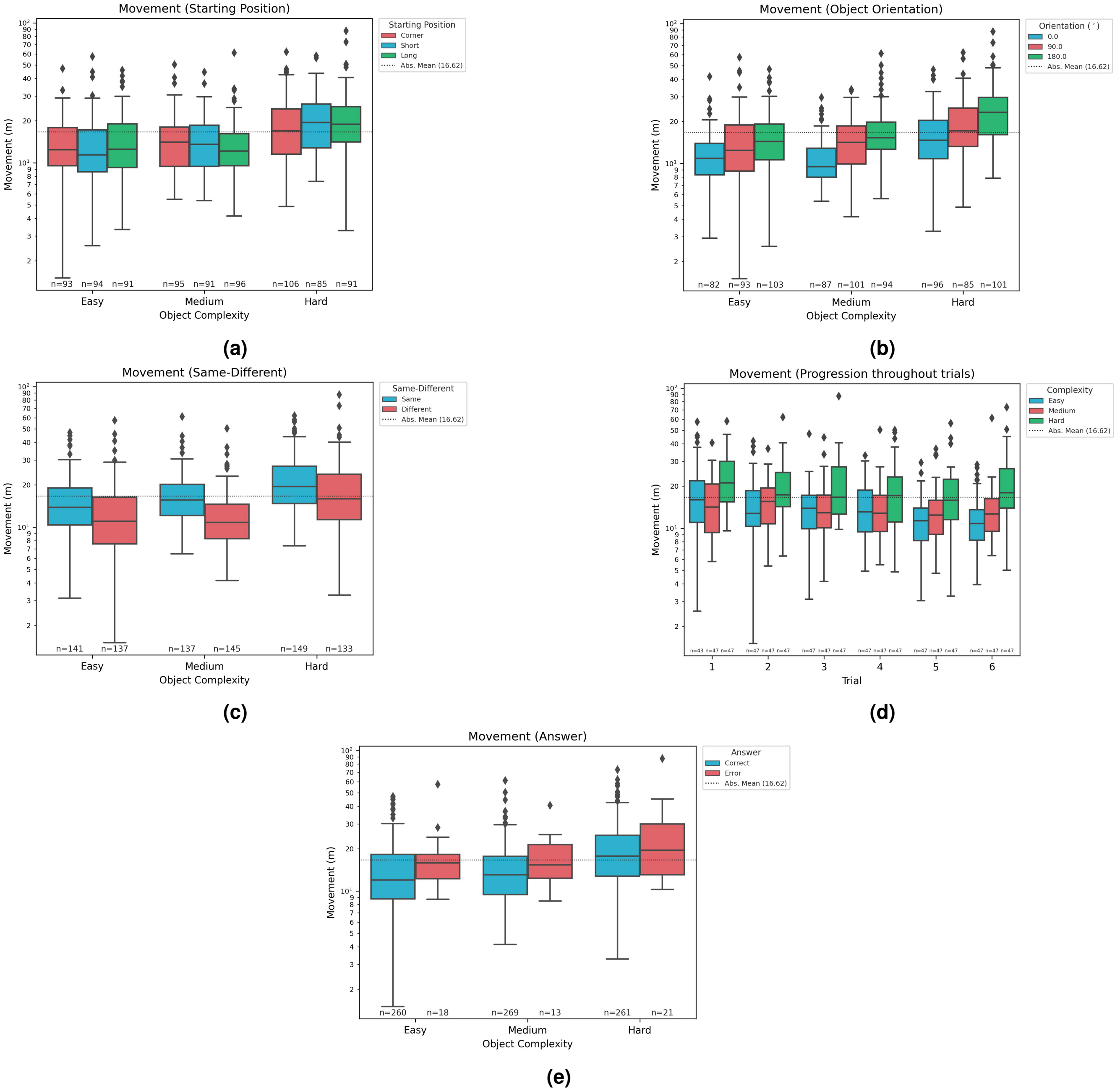

Figure S4. Head Movement against different experimental variables. **(a)** Starting position. We found no relationship between amount of head movement and starting position. **(b)** Object Orientation. A clear trend can be observed between the amount of head movement and the amount of orientational difference; at 0° the least amount of movement was required, at 90° an increase of 2-5m on average is recorded, and at 180° an additional increase of 1-5m across all complexity classes is recorded. **(c)** Sameness. Aligned with the number of fixations, response time and accuracy, the amount of movement necessary is greater for the same object pairings across all complexity levels. For different cases, the increased upper and lower quartiles indicate that more uncertainty across different subjects in how to approach this case was involved. **(d)** Across trials. Visualizes the effect of the progression through trials. A significant reduction in head movement is noticable over the course of the trials. This means that participants show a learning effect in the sense that they execute a strategy with less head movement as the experiment progresses. For C_m and C_h cases, a trend is not visible directly, but for the C_e cases, it is. C_h cases start off at the first trial with just above 20m and drop to the absolute mean value of 16.62m and stay steady, marginally falling below and exceeding it repetitively; similarly, for the C_m case, where no learning trend can be observed. However, the C_e case, while noticing a slight up-trend for the second trial, consecutively decreases from about 16m down to about 10m, which is the equivalent of an improvement of 37.5%. Lastly, **(e)** shows the effect of correct/error responses. However, there exists a significant effect on the correctness of the answer and head movement. Error responses were accompanied by significantly more head movement.

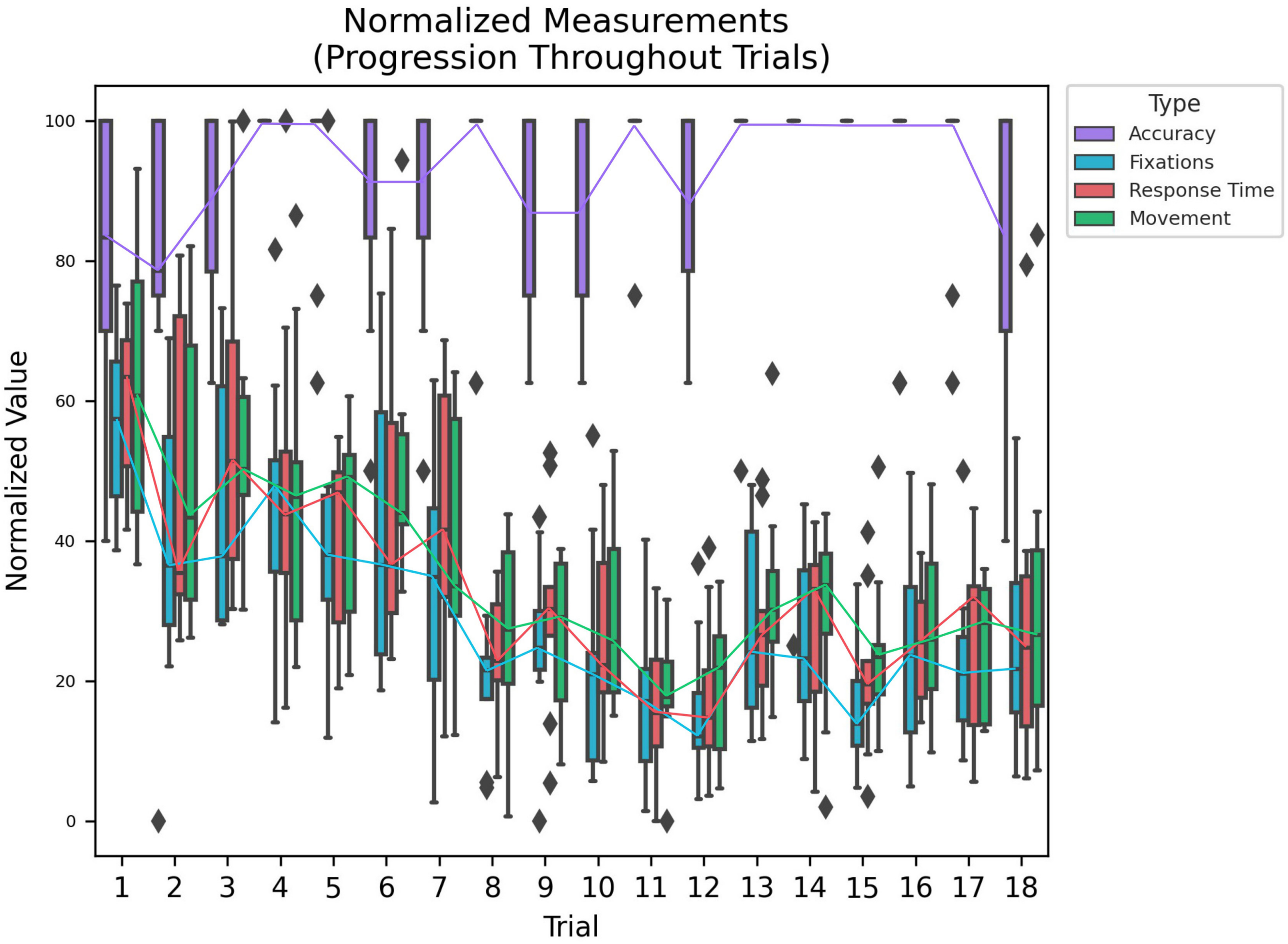

Figure S5. A plot of measured normalized variables (accuracy, fixations, response time and movement) with respect to trial number. It is easily seen that every measurement decreases significantly over the course of the trials except for accuracy.

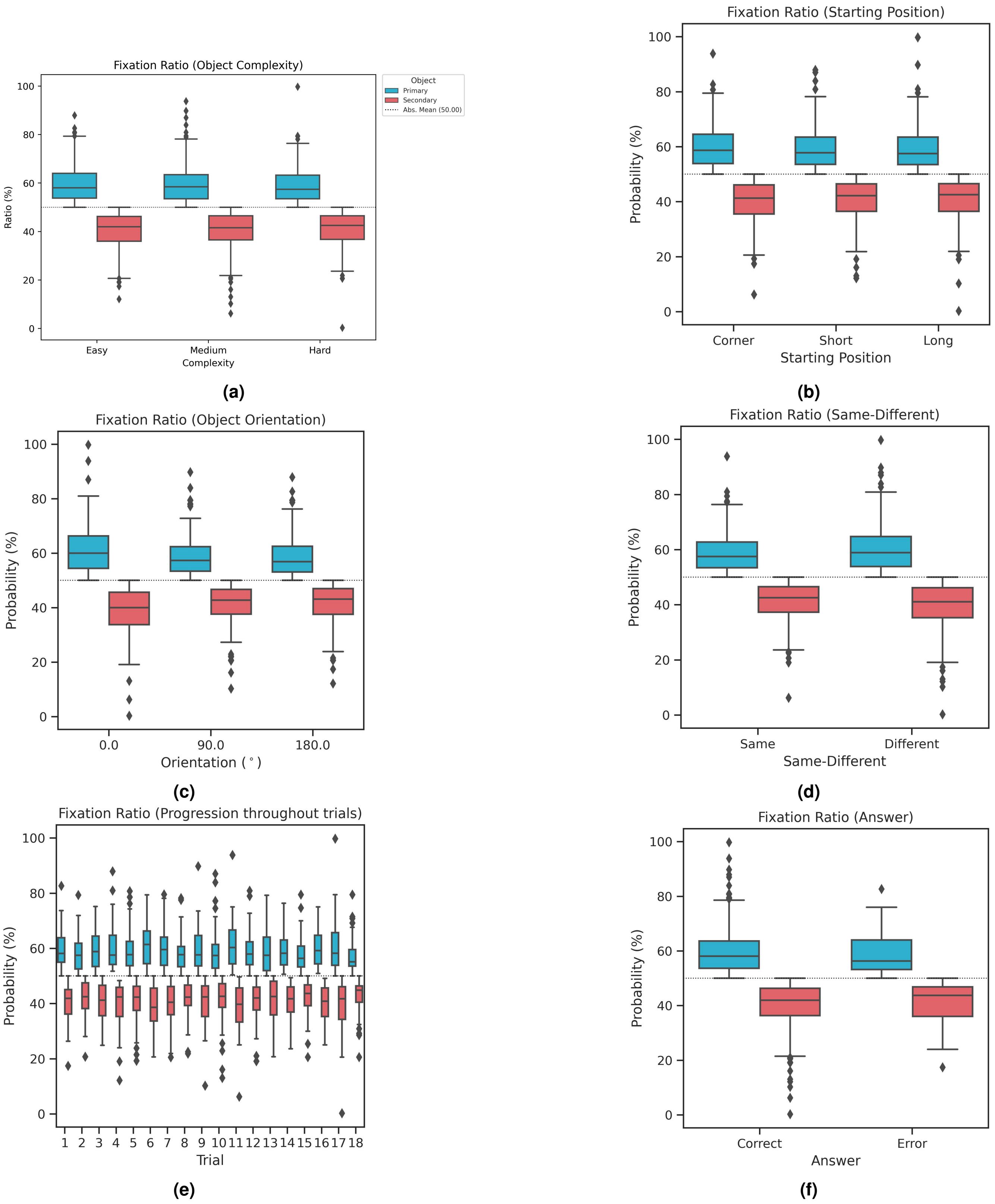

Figure S6. Ratio of fixations between both objects; **(a)** Object Complexity (this legend applies to all figures here), **(b)** Starting position, **(c)** Object Orientation, **(d)** Sameness, **(e)** Progression throughout trials, **(f)** Correct/Error Answer. We looked at the total number of fixations for either object. The object with the most fixations is considered the primary object, and the object with fewer or the same number of fixations is the secondary object. Interestingly, subjects tend to choose a primary and secondary object. On average, the primary object accounted for 59.53% of fixations, and the secondary object for 40.47%. However, none of the experimental variables have a significant effect on the fixation ratio.

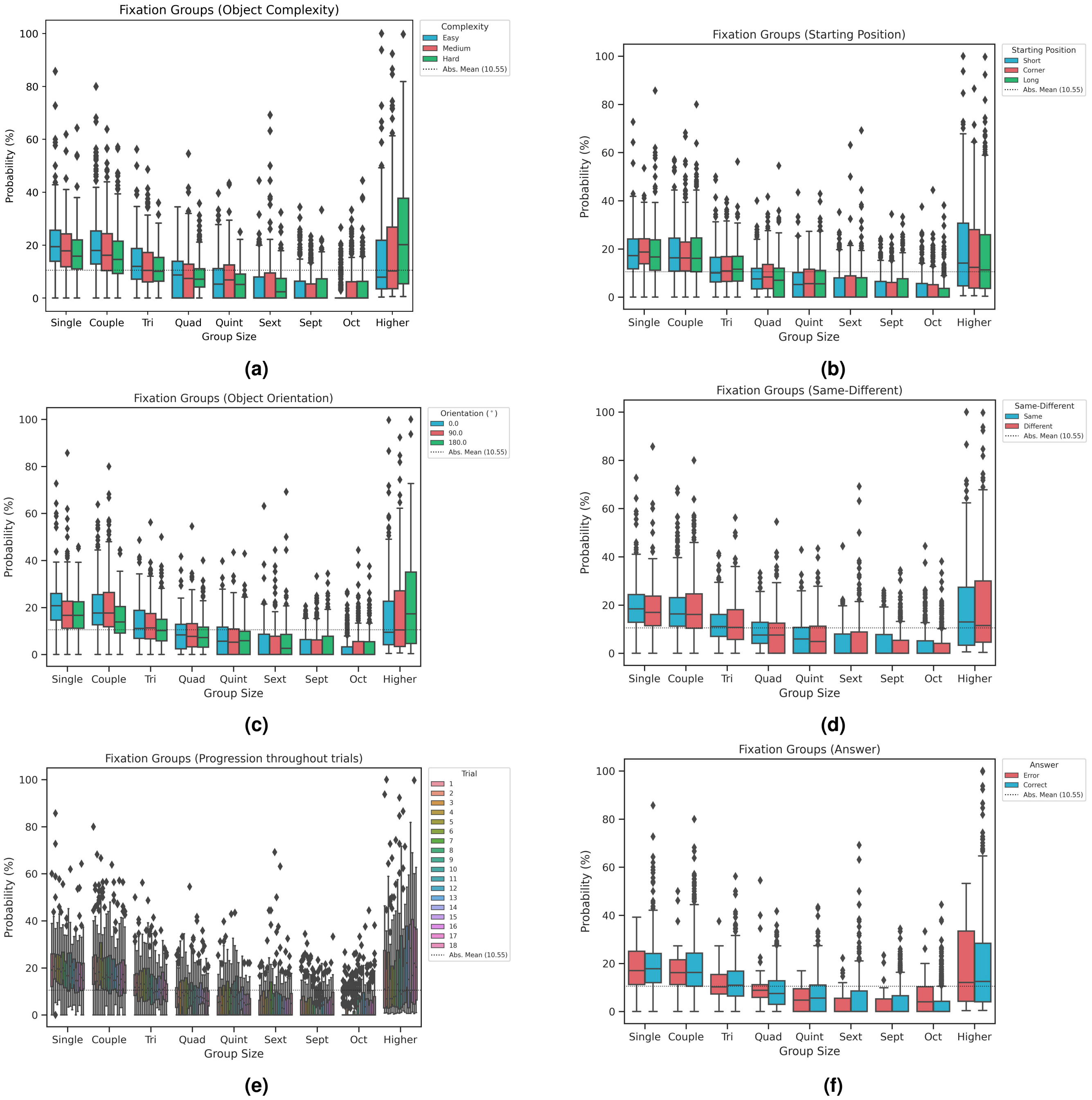

Figure S7. The result of the analysis of fixation groupings, i.e., the number of fixations on one object before changing focus to the other object. On average, 18.7% ($\sigma = 10.01\%$) are fixations that change focus between each object every time. **(a)** The object complexity has a significant effect on single, couple, triple, quintuple, octuple and higher fixation groupings. Notably, for single, couple, and triple, the probability of occurrence significantly decreases with increasing object complexity. While for octuple and higher groupings, the opposite is true – their occurrence increases with increasing object complexity. **(c)** The object orientation has a significant effect on single and couple groupings as this is the dominant method for object orientation 0° and decreases steadily with increasing object orientation. Similar to object complexity, larger fixation groupings are affected by object orientation as well. Specifically, septuple and higher groupings occur more frequently with increasing object orientation. **(d)** The sameness of the object had largely no significant effect on fixation groups. Only single and septuple groups are more significantly used for the same objects than different ones. **(e)** As trials advanced, subjects used single groupings progressively less. A similar trend is observed for couple, triple, quadruple and quintuple groups – none are significant, however. Larger groupings see an increase in probability as trials proceed. Notably, only a few sparse data points are recorded for octuple pairings up to trial 8. Octuple pairings are more regularly seen for trials 9-19. **(f)** The correctness of the answer significantly correlated with single and septuple pairs. Single and septuple fixation groups are significantly used more for correct answers than error responses.

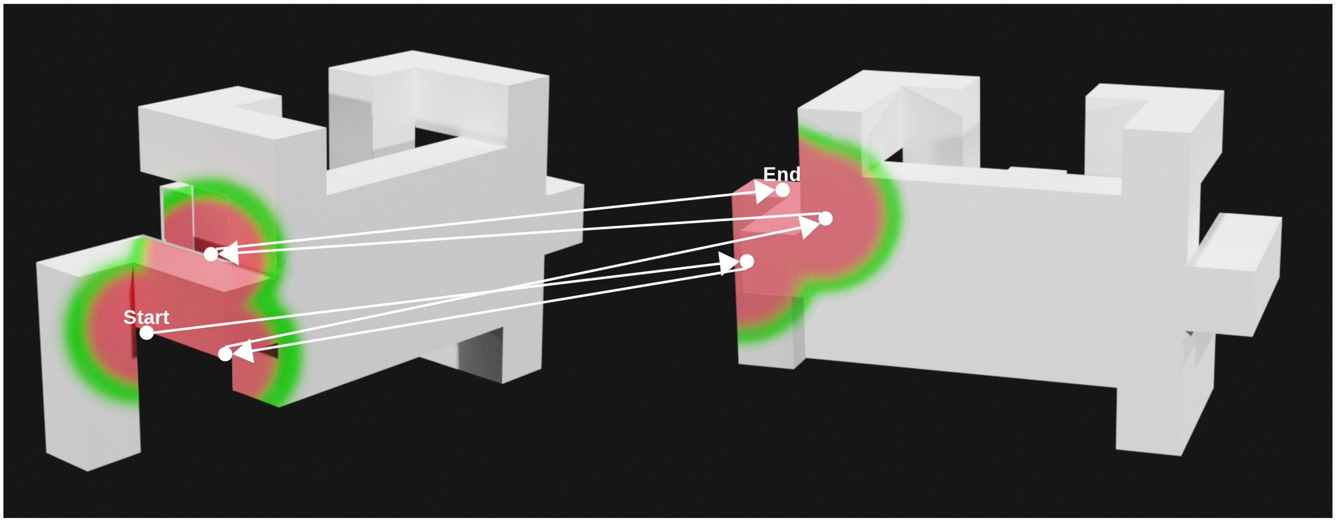

Figure S8. This is a drawing of an actual sequence of fixations between two objects. Both objects are shown in the orientation of their observation. The corresponding fixations are highlighted with red circles and a green border. Arrows point to and originate at the center of gaze. Further, the starting fixation is provided (annotated with “Start”), and the subsequent fixation is connected with an arrow. The alternating fixation ends at the fixation marked with “End.” The two objects are C_m; they are the same object, presented at 90° orientational difference. The mean accuracy for gaze fixations is 1.42°. Color encoded with uncertainty boundary in green. This illustration was created using *PESAO*[1] and *blender*[2].

DV \IV	Complexity	Starting Pos.	Sameness	Obj. Orientation	Trial
**Accuracy**	1.656	0.875	0.352	0.875	1.656
**Response Time**	0.002*	1.656	0.0044*	0.0019*	0.0018*
**Fixations**	0.0017*	1.29	0.0684	0.0016*	0.049*
**Movement**	0.0015*	1.656	0.0014*	0.0013*	0.0012*

Table 1. In this table is an additional analysis reported using one-way repeated-measures ANOVA with adjusted p-values using the Holm-Bonferroni method[3]. Values meeting the significance threshold ($p < 0.05$) are marked with an asterisk. The results are similar, with the only difference being that the sameness of objects does not significantly affect accuracy and number of fixations and that the object orientation does not significantly affect accuracy.

References

1. Solbach, M. D. & Tsotsos, J. K. PESAO: Psychophysical Experimental Setup for Active Observers. In *arXiv preprint arXiv:2009.09933*, 1–20 (2020).
2. Blender. Blender - a 3D modelling and rendering package. Version 3.3. https://www.blender.org (2022).
3. Holm, S. A simple sequentially rejective multiple test procedure. *Scand. journal statistics* 65–70 (1979).
